# Multidimensional predictive model for assessing clinical activity in thyroid eye disease

**DOI:** 10.3389/fmed.2025.1623286

**Published:** 2025-07-08

**Authors:** Yang Li, Guang-Hong Zhang, Man Tian, Chuan Hua, Jian-Ping Zhai, Yan-Qiong He, Xin-He Zuo

**Affiliations:** ^1^Thyroid Center of Hubei Provincial Hospital of Traditional Chinese Medicine, Wuhan, China; ^2^Hubei Shizhen Laboratory, Wuhan, China; ^3^Affiliated Hospital of Hubei University of Chinese Medicine, Wuhan, China; ^4^Department of Ophthalmology, Hubei Provincial Hospital of Traditional Chinese Medicine, Wuhan, China; ^5^Hubei Key Laboratory of The Theory and Application Research of Liver and Kidney in Traditional Chinese Medicine, Department of Hepatology, Hubei Provincial Hospital of Traditional Chinese Medicine, Wuhan, China; ^6^Department of Nuclear Medicine, Hubei Provincial Hospital of Traditional Chinese Medicine, Wuhan, China

**Keywords:** thyroid eye disease, SPECT/CT, clinical activity score, serum biomarkers, multidimensional model

## Abstract

**Objective:**

Thyroid eye disease (TED) is an autoimmune disorder with complex inflammatory activity that remains challenging to assess accurately. Current method, mainly the Clinical Activity Score (CAS), exhibits limitations in objectivity and comprehensiveness. This study aimed to develop a multidimensional predictive model integrating clinical parameters, SPECT/CT imaging data, and serum biomarkers, to improve TED activity evaluation.

**Methods:**

This retrospective research included 36 TED patients (72 eyes) diagnosed by EUGOGO criteria who underwent SPECT/CT examination. The Clinical Activity Score (CAS) was used to evaluate inflammatory activity. Variables with significant associations with CAS-defined activity were identified using univariate analysis, and Bayesian shrinkage regression (BSR) and the least absolute shrinkage and selection operator (LASSO) were utilized for variable selection in the primary cohort. Predictive models were constructed and evaluated using receiver operating characteristic (ROC) curves (internally validated via five-fold cross-validation), decision curve analysis (DCA), and calibration curves.

**Results:**

Five predictive models were constructed. The comprehensive Model 4, combining clinical, imaging [EX, maximal SPECT/CT uptake ratio (URmax)], and serum biomarkers (TRAb, RBC), achieved superior diagnostic accuracy (AUC: 91.18%; sensitivity: 0.91; specificity: 0.86). Model 5, retaining variables significant in univariate and multivariate analyses, demonstrated robust performance (AUC: 85.97%) with superior stability during cross-validation (ROC mean: 0.8417). Key predictors included male sex (OR = 11.74), TRAb levels, EX, URmax, and RBC count. SPECT/CT-derived URmax correlated strongly with disease activity, while serum biomarkers complemented imaging limitations.

**Conclusion:**

Multidimensional integration of clinical, imaging, and biomarker data significantly enhances TED activity evaluation compared to single-modality approaches. The multidimensional model offers superior diagnostic accuracy, addressing the limitations of conventional methods. These findings advocate for a holistic approach in TED management.

## Introduction

Thyroid eye disease (TED), which is also referred to as Graves’ orbitopathy (GO) or thyroid-associated ophthalmopathy (TAO), manifests as an autoimmune disorder involving inflammation and fibrosis of the orbital tissues (1, 2). As a frequent extra-thyroidal manifestation of Graves’ disease, TED exhibits a symptom spectrum spanning mild conjunctival inflammation to severe orbital complications including proptosis, diplopia, and optic nerve impairment (3). The prevalence of TED in Graves’ disease patients ranges from 25 to 50%, and is even higher in uncontrolled hyperthyroidism populations (4).

Accurate assessment of TED activity is critical for guiding treatment decisions. The Clinical Activity Score (CAS), a 7-point scale based on clinical signs (e.g., eyelid swelling, conjunctival redness), is widely used to evaluate inflammatory activity (5). However, CAS exhibits limitations in reproducibility and subclinical inflammation detection, and its results are highly dependent on the doctor’s sensitivity. A cohort study in Asian populations demonstrated that 38% with moderate-to-severe and 10% with vision-threatening TED had CAS <3, suggesting potential underestimation of disease activity (6). These discrepancies highlight the need for objective methods.

Single-photon emission computed tomography/computed tomography (SPECT/CT) has improved the quantification of orbital inflammation (7). By measuring the uptake ratio (UR) of radiotracers ^99m^Tc-DTPA in extraocular muscles relative to the occipital lobe, SPECT/CT provides semi-quantitative data correlating with disease severity (8). A multimodal radiomic model incorporating ^99m^Tc-DTPA SPECT/CT imaging demonstrated statistically significant precision in assessing TED clinical activity (9). Despite these advances, SPECT/CT has its limitations: it primarily reflects localized muscular metabolic activity and fails to capture systemic immune dysregulation, which plays a central role in TED pathogenesis.

Emerging evidence suggests that systemic biomarkers could complement imaging modalities (10–12). For instance, thyrotrophin receptor antibody (TRAb) levels correlate with TED progression and recurrence (4). Integrating clinical biomarkers with imaging data may enhance diagnostic precision. This study aims to construct a multidimensional model combining clinical data, SPECT/CT data, and serum biomarkers to improve the accuracy of TED activity evaluation.

## Methods

### Study design and subjects

**Figure 1:**
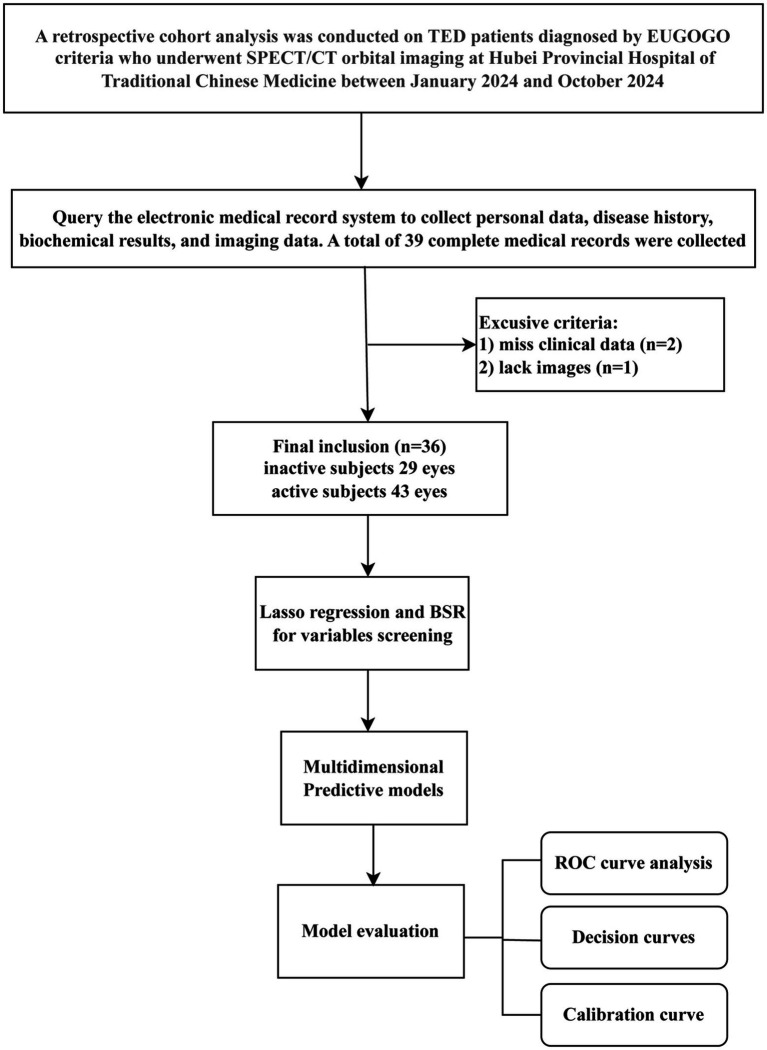
The comprehensive workflow of this research.

This retrospective study included 36 TED patients (72 eyes), diagnosed by EUGOGO criteria (13), that were undertook SPECT/CT examination at Hubei Provincial Hospital of Traditional Chinese Medicine between January 2024 and October 2024. The inclusion criteria for TED patient: (1) age >18 years, (2) sufficient image quality, and (3) without systemic glucocorticoid treatment for at least 3 months prior to the study. The exclusion criteria: (1) non-thyroid systemic comorbidities, (2) prior ocular surgery/trauma, and (3) non-TED ophthalmic conditions. Ethical clearance (Approval No. HBZY2024-C04-01) was obtained from Hubei Provincial Hospital of Traditional Chinese Medicine’s Ethics Committee, following the Declaration of Helsinki guidelines. The comprehensive workflow of this research was shown in Figure 1.

### Clinical and systemic measurements

The clinical information, including sex, age, body mass index (BMI), systolic blood pressure (SBP), diastolic blood pressure (DBP), was recorded. Clinical activity assessment was conducted on a per-eye basis using the Clinical Activity Score (CAS) criteria (14). Thyroid function assessment was performed using electrochemiluminescent immunoassays. Concentrations of serum free triiodothyronine (FT3), free thyroxine (FT4), thyroid-stimulating hormone (TSH), thyroglobulin antibodies (TGAb), and thyroid peroxidase antibodies (TPOAb) were analyzed on a SIEMENS Atellica IM analyzer (Siemens Healthineers, Germany) using manufacturer-supplied kits (Ref. Nos. 10995584, 10995588, 10995704, 11201761, and 10995467, respectively). Thyroid-stimulating hormone receptor antibodies (TRAb) were measured on a Roche cobase 411 analyzer (Roche Diagnostics, Germany) using the Immulite TRAb kit (Ref. No. 08496609190). All assays followed standardized protocols per kit instructions: 50 μL serum samples were incubated with paramagnetic particles coated with capture antibodies/antigens, followed by chemiluminescent substrate addition. Signal detection was proportional to analyte concentration, calibrated against traceable reference materials. Serum biomarkers examination included a complete blood count, lipid profile, and the calculation of neutrophil-to-lymphocyte ratio (NLR), platelet-to-lymphocyte ratio (PLR), and systemic immune-inflammatory index (SII) (platelet count × neutrophil count/lymphocyte count).

### Ophthalmic examination by ^99m^Tc-DTPA SPECT/CT scan

^99m^Tc-DTPA at a dose of 370–555 MBq was administered through standardized antecubital venous access. Subsequent SPECT/CT scans were obtained 30–45 min later by a dual-head gamma camera system (Discovery NM/CT 670 Pro, GE Healthcare, USA) as described in previous report (15). The uptake ratio (UR) of extraocular muscles of each eye included was calculated by two senior nuclear medicine physicians blinded to the grouping. The degree of the exophthalmos was evaluated on the basis of SPECT/CT. The straight line joining the anterior tips of the zygomatic bones was the line of reference. A distance greater than 20 mm from the reference line to the anterior border of the eye was considered diagnostic of exophthalmos (EX) (8).

### Statistical analysis

Spearman’s correlation was used to assess associations between variables and CAS-defined activity. All variables were subjected to univariate analysis, and those with statistically significant associations (*p* < 0.05) were included in the subsequent multivariate model. To select variables in the primary cohort and avoid over-fitting or under-fitting, two multivariate regression methods—Bayesian shrinkage regression (BSR) and least absolute shrinkage and selection operator (LASSO)—were employed. For BSR, variable selection was guided by the Bayesian information criterion (BIC). Predictive models were constructed and evaluated using receiver operating characteristic (ROC) curves, assessed through internal five-fold cross-validation for stability, followed by decision curve analysis (DCA), and calibration curves. All the analyses were conducted using software packages R (version 4.2.2, The R Foundation, http://www.R-project.org) and Free Statistic software (version 2.1, Beijing, China).

## Results

### Basic demographic data and clinical characteristics

**Figure 2:**
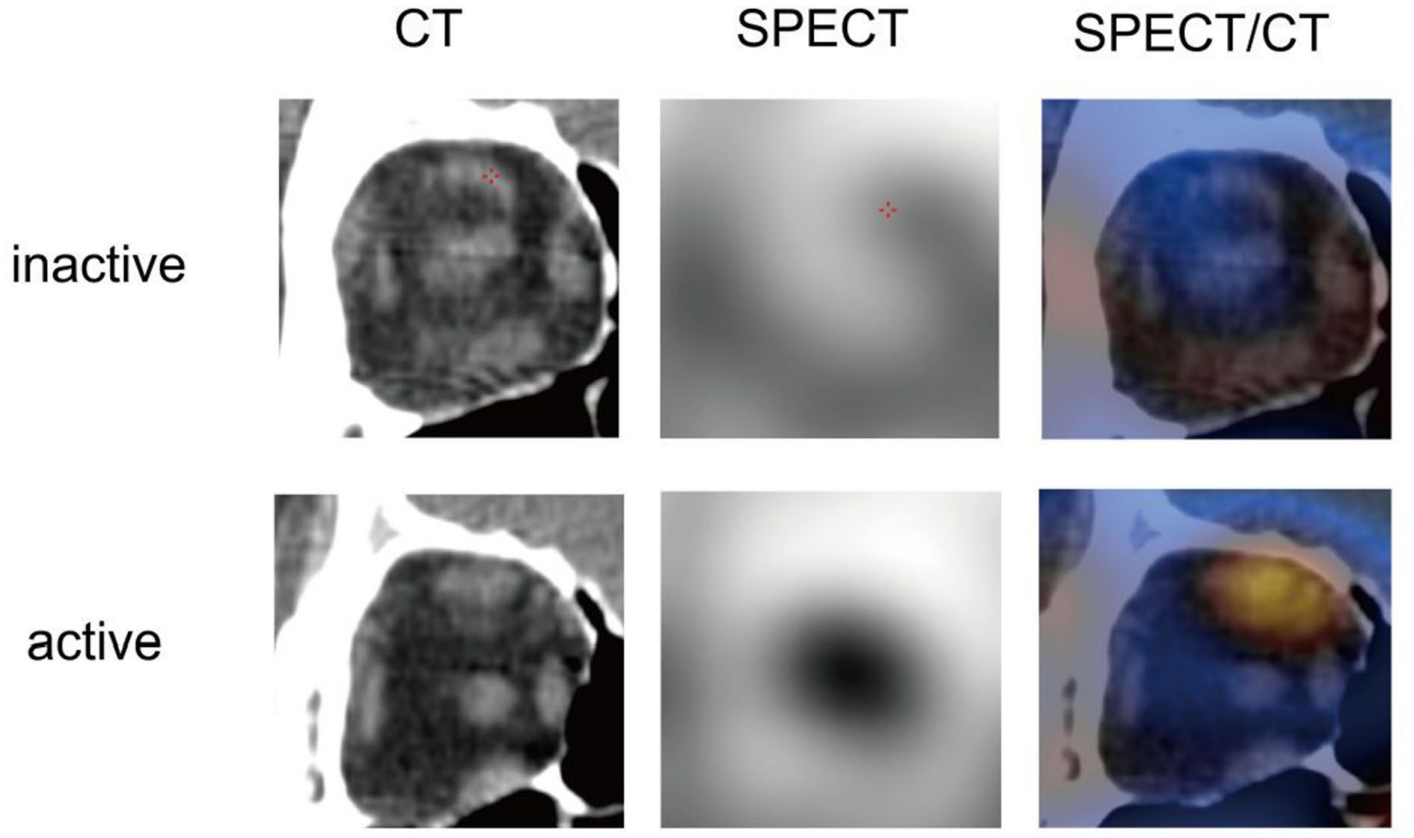
Representative unilateral radiographic manifestations of active and inactive TED defined by CAS.

A total of 29 inactive eyes and 43 active eyes of TED patients were included in this study. The demographic data and clinical characteristics were depicted in Table 1. Significant differences between the two groups were observed in sex, SBP, EX, URmax, TRAb, RBC, and LDL-C (all *p <* 0.05). Representative unilateral radiographic manifestations of active and inactive TED defined by CAS were shown in Figure 2. The active TED exhibited increased radionuclide uptake in orbital tissues, including the extraocular muscles, lacrimal glands, and paranasal sinuses.

**Table 1:** Comparison of basic demographic data and clinical characteristics between active and inactive TED patients defined by CAS.

Variables	Total (*n* = 72)	Inactive (*n* = 29)	Active (*n* = 43)	*p*
Sex, *n* (%)				<0.001
Female	50 (69.4)	27 (93.1)	23 (53.5)	
Male	22 (30.6)	2 (6.9)	20 (46.5)	
Age, mean ± SD	42.4 ± 11.5	40.0 ± 9.2	44.0 ± 12.7	0.149
BMI, mean ± SD	22.4 ± 3.1	21.5 ± 3.0	22.9 ± 3.1	0.06
SBP, mean ± SD	123.2 ± 15.5	117.6 ± 10.3	127.0 ± 17.3	0.01
DBP, mean ± SD	86.1 ± 9.5	85.1 ± 7.5	86.8 ± 10.7	0.465
EX, mean ± SD	19.3 ± 2.6	18.1 ± 2.1	20.2 ± 2.5	<0.001
URmax, mean ± SD	5.2 ± 1.4	4.7 ± 1.3	5.5 ± 1.4	0.022
FT3, median (IQR)	5.8 (4.8, 9.3)	5.5 (4.7, 9.7)	6.3 (5.3, 8.8)	0.228
FT4, median (IQR)	15.8 (13.9, 24.9)	15.3 (12.6, 24.1)	16.1 (14.0, 23.3)	0.329
TSH, median (IQR)	0.1 (0.0, 1.9)	0.0 (0.0, 2.4)	0.2 (0.0, 1.8)	0.945
TGAb, median (IQR)	8.2 (1.3, 103.0)	12.2 (1.3, 758.5)	4.1 (1.3, 95.1)	0.417
TPOAb, median (IQR)	790.5 (42.3, 1300.0)	226.7 (46.2, 1300.0)	852.8 (45.0, 1300.0)	0.893
TRAb, median (IQR)	10.1 (4.7, 29.4)	5.6 (1.7, 14.1)	16.7 (4.8, 30.0)	0.042
CRP, median (IQR)	0.8 (0.4, 1.8)	0.5 (0.3, 1.6)	1.0 (0.4, 2.1)	0.151
WBC, mean ± SD	5.7 ± 1.2	5.7 ± 1.2	5.8 ± 1.2	0.636
RBC, mean ± SD	4.9 ± 0.7	4.6 ± 0.4	5.1 ± 0.8	0.009
PLT, mean ± SD	237.8 ± 53.5	236.2 ± 49.1	238.8 ± 56.8	0.84
NEU, mean ± SD	3.5 ± 1.2	3.4 ± 1.1	3.5 ± 1.2	0.546
LYM, mean ± SD	1.8 ± 0.7	1.8 ± 0.3	1.8 ± 0.8	0.561
NLR, mean ± SD	2.2 ± 1.1	1.9 ± 0.8	2.4 ± 1.3	0.061
PLR, mean ± SD	142.7 ± 41.1	132.2 ± 35.7	149.7 ± 43.4	0.076
SII, mean ± SD	503.8 ± 247.0	449.3 ± 220.2	540.5 ± 259.5	0.125
TC, mean ± SD	4.7 ± 1.0	4.5 ± 0.7	4.8 ± 1.1	0.117
TG, mean ± SD	1.3 ± 0.6	1.1 ± 0.5	1.4 ± 0.7	0.125
HDL-C, mean ± SD	1.3 ± 0.3	1.4 ± 0.2	1.3 ± 0.3	0.064
LDL-C, mean ± SD	2.6 ± 0.8	2.3 ± 0.6	2.8 ± 0.9	0.017

TED, thyroid eye disease; CAS, Clinical Activity Score; BMI, body mass index; SBP, systolic blood pressure, DBP, diastolic blood pressure; EX, exophthalmos; URmax, the highest uptake ratio of each eye; FT3, free triiodothyronine; FT4, free thyroxine; TSH, thyroid stimulating hormone; TGAb, thyroglobulin antibody; TPOAb, thyroid peroxidase antibody; TRAb, thyroid-stimulating hormone receptor antibody; CRP, C reactive protein; WBC, white blood cell; RBC, red blood cell; PLT, platelet; NEU, neutrophil; LYM, lymphocyte; NLR, neutrophil-to-lymphocyte ratio; PLR, platelet-to-lymphocyte ratio; SII, systemic immune-inflammatory index; TC, total cholesterol; TG, triglyceride; HDL-C, high-density lipoprotein cholesterol; LDL-C, low density lipoprotein cholesterol.

### Univariate analysis of variables associated with CAS-defined activity

Univariate analysis revealed that sex, SBP, EX, URmax, TRAb, and RBC were significantly associated with CAS-defined disease activity (Table 2). Males showed a higher odds ratio (OR) for clinical activity compared to females (OR = 11.74, *p* = 0.002). SBP (OR = 1.05, *p* = 0.015), EX (OR = 1.49, *p* = 0.002), URmax (OR = 1.55, *p* = 0.028), TRAb (OR = 1.04, *p* = 0.038), and RBC (OR = 3.28, *p* = 0.013) were positively correlated with clinical activity. In contrast, no significant associations were found for age, BMI, DBP, FT3, FT4, TSH, CRP, WBC, PLT, NEU, LYM, NLR, PLR, SII, TGAb, and TPOAb (all *p* > 0.05) (Table 2).

**Table 2:** Univariate analysis of variables associated with CAS-defined activity.

Variable	OR 95% CI	*p*-value
Sex		
Female	1 (reference)	
Male	11.74 (2.48–55.64)	0.002
Age	1.03 (0.99–1.08)	0.151
BMI	1.18 (0.99–1.4)	0.065
SBP	1.05 (1.01–1.09)	0.015
DBP	1.02 (0.97–1.07)	0.46
EX	1.49 (1.16–1.92)	0.002
URmax	1.55 (1.05–2.31)	0.028
FT3	1.07 (0.99–1.17)	0.096
FT4	1.03 (0.99–1.06)	0.168
TSH	0.94 (0.86–1.03)	0.195
TGAb	1 (1–1)	0.082
TPOAb	1 (1–1)	0.636
TRAb	1.04 (1–1.08)	0.038
CRP	1.13 (0.87–1.46)	0.355
WBC	1.1 (0.74–1.65)	0.631
RBC	3.28 (1.28–8.42)	0.013
PLT	1 (0.99–1.01)	0.837
NEU	1.14 (0.75–1.72)	0.541
LYM	0.81 (0.39–1.66)	0.56
NLR	1.6 (0.96–2.64)	0.069
PLR	1.01 (1–1.02)	0.079
SII	1 (1–1)	0.128

### Variables selection using the BSR and the LASSO

**Figure 3:**
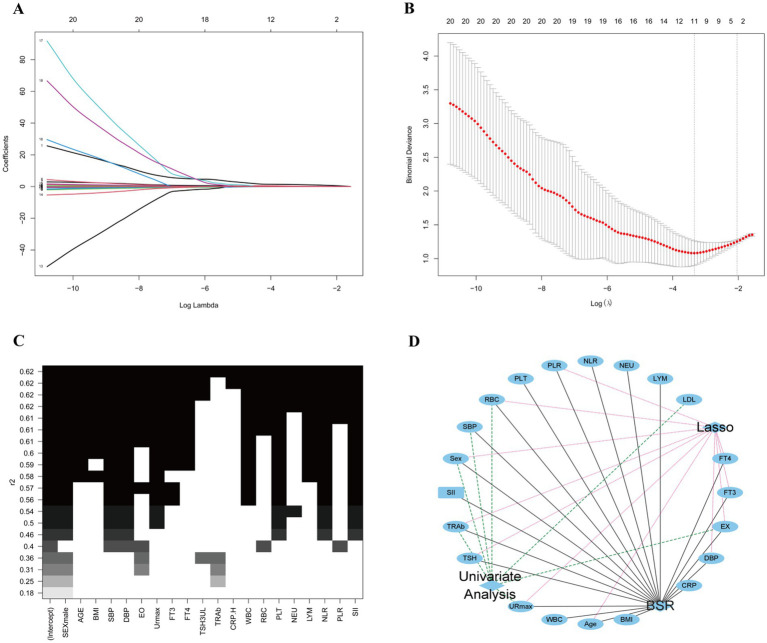
Variables for predicting model screening results. **(A,B)** Variables screening based on the LASSO regression. **(C)** Variables screening based on the BSR. **(D)** Variables selected from both univariate and regression analyses with statistical significance.

The combined application of BSR and LASSO identified sex, age, DBP, EX, URmax, FT3, FT4, TSH, TRAb, RBC, and PLR as significant predictors (Figures 3A–C). Five predictive models were constructed:

(i) Model 1: exclusively incorporated imaging data URmax and EX.
(ii) Model 2: integrated clinical characteristics (sex, age, and DBP) with imaging data (URmax and EX).
(iii) Model 3: integrated clinical characteristics (sex, age, and DBP) with blood markers (FT3, FT4, TSH, TRAb, RBC, and PLR).
(iv) Model 4: comprehensively combined clinical characteristic, imaging data, and blood markers.
(v) Model 5: selected variables with statistical significance from both univariate and regression analyses (sex, EX, URmax, TRAb, and RBC) (Figure 3D).

### ROC curve analysis for multidimensional predictive models

**Figure 4:**
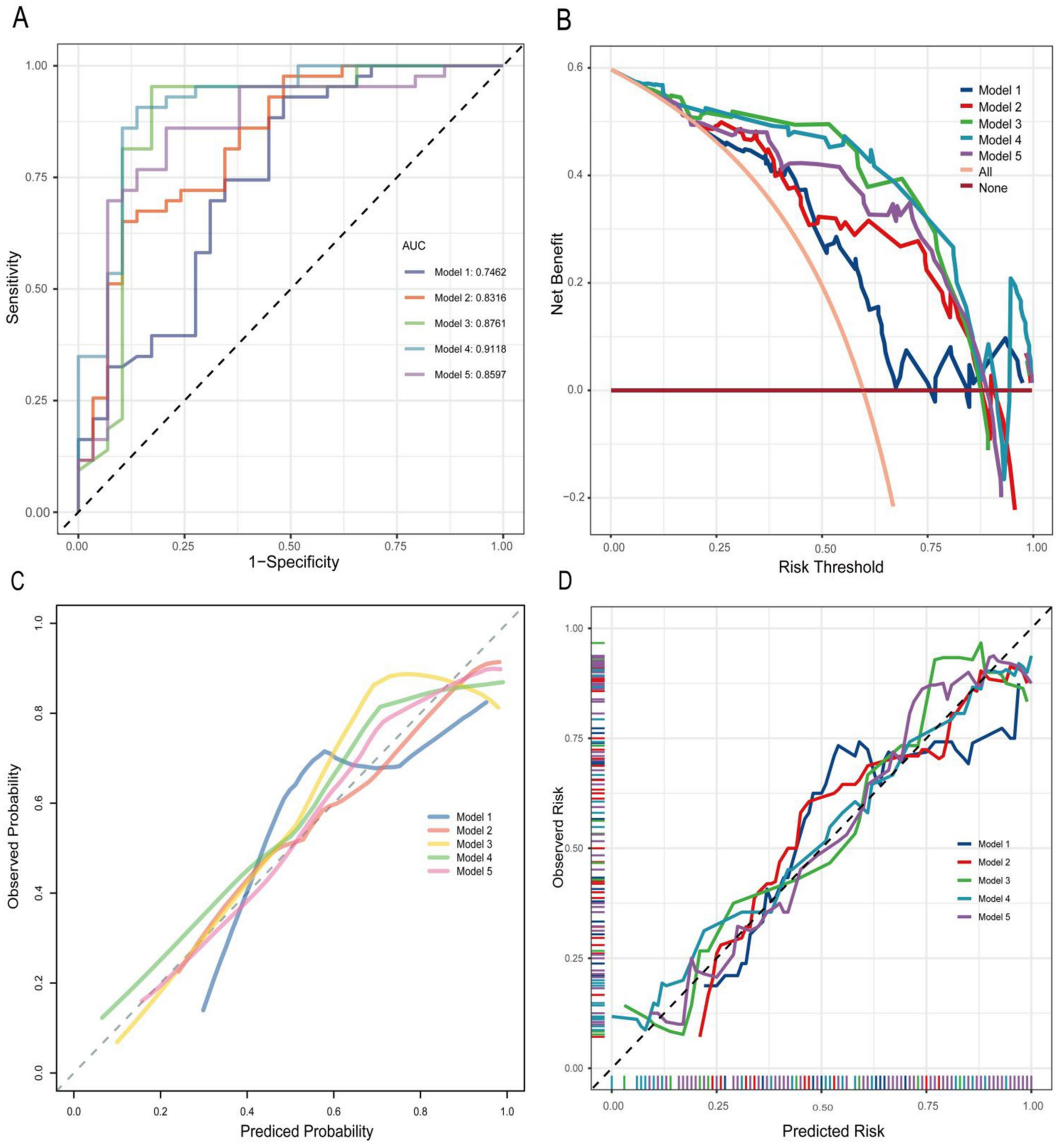
**(A)** ROC curve of multidimensional predictive models. AUC, area under the curve. **(B)** DCA of multidimensional predictive models. **(C,D)** Calibration curve of multidimensional predictive models.

ROC analysis demonstrated varying diagnostic performance across models (Table 3 and Figure 4A). Model 4 achieved the highest discriminative power, with an AUC of 91.18% (95% CI: 83.82–98.54%) and an optimal threshold of 0.55, achieving balanced sensitivity (0.91), specificity (0.86), and accuracy (0.89), alongside strong predictive values (PPV: 0.91, NPV: 0.86). Model 3 ranked second in AUC (87.61, 95% CI: 77.49–97.73%) with the highest sensitivity (0.95) at a threshold of 0.48, moderate specificity (0.83) and accuracy (0.90). In contrast, Model 2 prioritized specificity (0.90) over sensitivity (0.65) at a high threshold (0.71), yielding the highest PPV (0.90) but lower accuracy (0.75). Model 5 exhibited an AUC of 85.97% (95% CI: 76.35–95.59%) at a threshold of 0.51, with balanced sensitivity (0.86) and specificity (0.79). Model 1 showed the weakest performance (AUC: 74.62, 95% CI: 62.55–86.69%), characterized by high sensitivity (0.93) but low specificity (0.52).

**Table 3:** ROC parameters of multidimensional predictive models.

ROC	AUC	95% CI	Best threshold	Specificity	Sensitivity	Accuracy	NPV	PPV
Model 1	74.62%	62.55%–86.69%	0.42	0.52	0.93	0.76	0.83	0.74
Model 2	83.16%	73.32%–93%	0.71	0.9	0.65	0.75	0.63	0.9
Model 3	87.61%	77.49%–97.73%	0.48	0.83	0.95	0.9	0.92	0.89
Model 4	91.18%	83.82%–98.54%	0.55	0.86	0.91	0.89	0.86	0.91
Model 5	85.97%	76.35%–95.59%	0.51	0.79	0.86	0.83	0.79	0.86

AUC, area under the curve; PPV, positive predictive value; NPV, negative predictive value.

**Figure 5:**
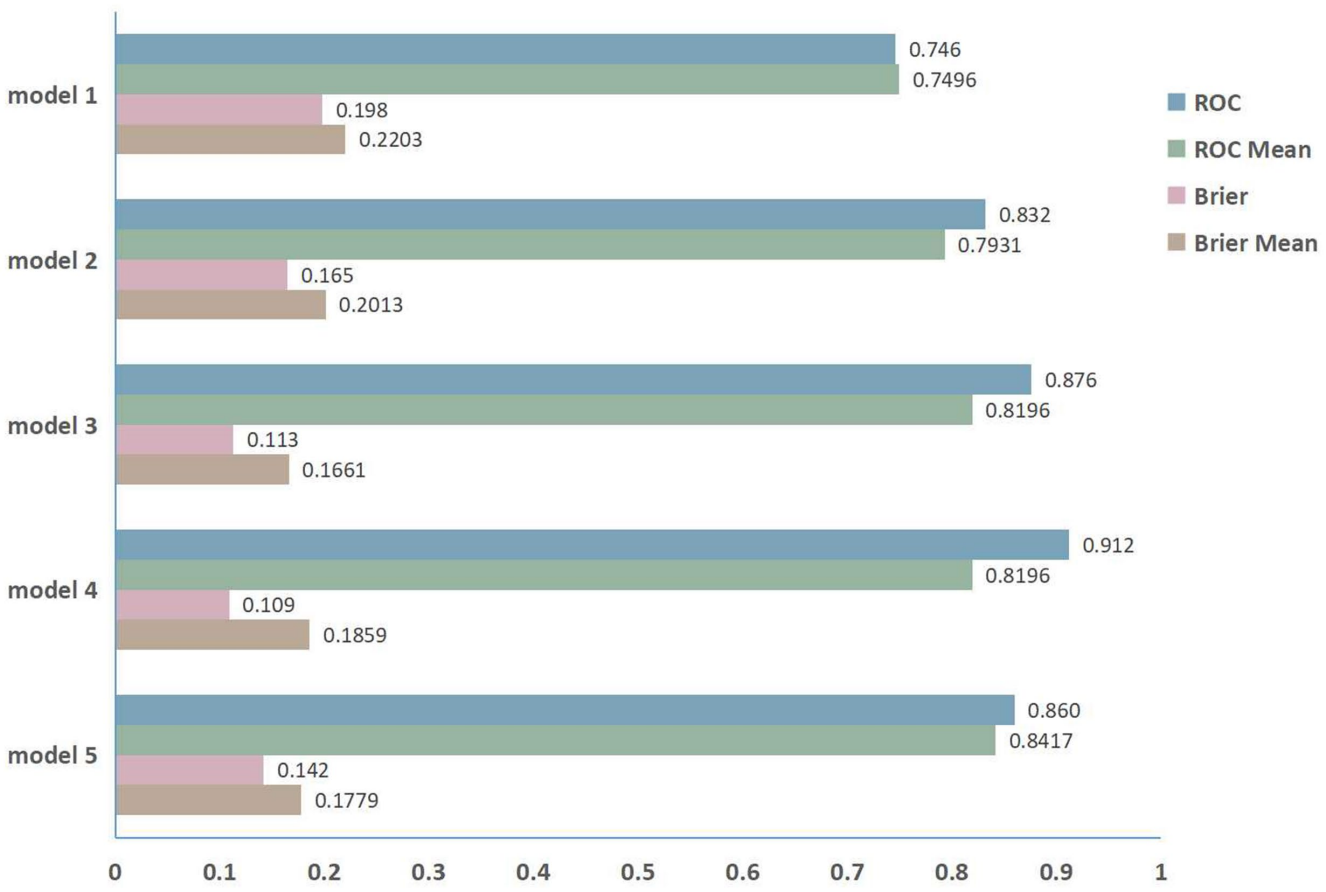
The five-fold cross-validation performance.

Using five-fold cross-validation to evaluate model stability and generalizability (Figure 5), we observed minimal divergence between the primary ROC (0.746) and cross-validated ROC mean (0.7496) for Model 1. Model 2 exhibited a moderate reduction (primary ROC: 0.832; ROC mean: 0.7931). Model 3 showed greater performance reductions (primary ROC: 0.876; ROC mean: 0.8196). Model 4, which incorporated the broadest variable set, yielded the largest discrepancy between primary and cross-validated performance (primary ROC: 0.912; ROC mean: 0.8196). Conversely, Model 5 maintained high consistency (primary ROC: 0.860; ROC mean: 0.8417), indicating superior stability. The mean Brier scores for Model 3, Model 4, and Model 5 were consistently below 0.2.

### DCA analysis for multidimensional predictive models

The DCA analysis of multidimensional predictive models was presented in Figure 4B. Model 4 demonstrated the highest net benefit across a broad range of probability thresholds, significantly outperforming the other models. Model 3 also showed a relatively high net benefit, but it was slightly lower than Model 4 in most cases. Model 2 had a moderate net benefit compared to other models. Model 5 exhibited a net benefit that was lower than Model 4 but higher than Model 1 across most probability thresholds. In contrast, Model 1 consistently exhibited the lowest net benefit across nearly all probability thresholds, indicating its limited effectiveness in this analysis.

### Calibration curve analysis for multidimensional predictive models

The calibration curve analysis for the multidimensional predictive models, as illustrated in Figures 4C,D, evaluated the alignment between predicted probabilities and actual outcomes across the five models. Model 4 exhibited the best calibration, with its curve closely following the line of identity (ideal calibration), indicating that its predicted probabilities align well with observed outcomes across different probability thresholds. Model 3 also revealed relatively good calibration, though with slight deviations from the ideal line in certain regions. Model 2 exhibited moderate calibration performance, with larger deviations from the ideal line compared to Models 3 and 4. Model 5 achieved moderate calibration, with minor deviations in both low and high probability extremes. In contrast, Model 1 had the poorest calibration, as its curve deviated significantly from the line of identity across most probability thresholds.

## Discussion

This study developed a multidimensional predictive model integrating clinical characteristics, SPECT/CT imaging data, and serum biomarkers to assess clinical activity in thyroid eye disease (TED). The results demonstrated that the comprehensive model (Model 4), which combined all three data dimensions, outperformed models relying on single or partial parameters, achieving an AUC of 91.18% with balanced sensitivity (0.91) and specificity (0.86). This highlights its potential utility in guiding clinical decision-making.

CAS, as an internationally recognized assessment of thyroid associated eye disease activity, has irreplaceable advantages such as simplicity, non-invasiveness, and low cost. However, due to its reliance on inspectors, CAS has been shown to have a certain degree of subjectivity and unreliable inter-observer variability (16). In our dataset, 8 of 72 eyes with a CAS score ≤2 exhibited orbital inflammatory edema and extraocular muscle involvement, with SPECT/CT-derived uptake ratio (UR) values exceeding 5.0. These findings suggest that exclusive reliance on CAS scores may fail to fully capture the underlying pathological processes of inflammatory activity. Thus, establishing a comprehensive assessment method that incorporates multiple factors is essential to enhance diagnostic accuracy.

Orbital SPECT/CT has incomparable advantages over orbital CT plain scan (9). SPECT/CT technology offers metabolic and functional insights into orbital tissues by measuring radioactive tracer uptake, thereby providing an intuitive reflection of orbital inflammatory activity (17). In accordance with previous studies (15, 18), our results showed that the orbital SPECT/CT UR in the active phase group were higher than those in the stable phase group, indicating that orbital SPECT/CT UR are meaningful for assessing the activity of TED patients. While SPECT/CT is effective for TED evaluation, it also has limitations compared to other imaging techniques like MRI, which excels in soft tissue resolution and multi-parametric imaging (18). Future research should explore combining SPECT/CT with other imaging modalities to leverage their complementary strengths and further enhance the accuracy of TED assessment.

SPECT/CT imaging effectively reflects the morphological and metabolic characteristics of local orbital tissues 22 (19). However, as it primarily captures cellular metabolic activity within the orbit, it may not fully represent systemic inflammatory processes or immune reactivity. Moreover, radionuclide physiological accumulation in the nasal sinus mucosa near the orbit and in tears can cause false-positive results in SPECT/CT imaging (20, 21). In our data, 18 of 72 eyes with UR values <5.0 showed CAS levels exceeding 3.0, highlighting the limitations of using UR as a sole indicator. TED disease activity is closely associated with clinical markers and blood biomarkers, such as TRAb, NLR, PLR, and SII, which play critical roles in disease progression and therapeutic response (22–25). This understanding has driven the evolution from traditional imaging-based approaches toward more integrative models that incorporate systemic health indicators. By combining SPECT/CT findings with serum markers such as TRAb, we can develop a more comprehensive assessment framework. This multimodal approach not only enhances the characterization of disease activity but also improves diagnostic accuracy in distinguishing active from inactive phases of TED, thereby optimizing clinical decision-making.

In the present study, univariate analysis identified sex, SBP, EX, URmax on SPECT/CT, TRAb levels, and RBC count as significant predictors of TED activity. Regarding sex differences, our results corroborate previous findings. A meta-analysis identified a significant positive correlation between male sex and active TED (26), and a large Chinese study reported a substantially higher prevalence of active-stage TED among males compared to females (27). Notably, our investigation demonstrated a markedly elevated risk of active TED in males (OR = 11.74), reflecting sex-related differences in immune regulation or genetic susceptibility, which needs further mechanistic exploration. Specifically regarding SBP, our findings confirm its association with TED activity. This observation aligns with previous research suggesting that abnormal blood pressure, including elevated SBP in TED patients, may impact retinal vascular perfusion. Furthermore, elevated pulse pressure has been proposed to affect retinal vasculature prior to the onset of mechanical compression factors (28). The precise relationship between blood pressure dysregulation and TED pathogenesis warrants further investigation. EX, a key clinical feature of TED and a critical indicator for evaluating treatment efficacy (29), was also significantly associated with clinical activity in our cohort, consistent with established literature. TRAb, a core biomarker in TED pathogenesis, consistently correlated with disease activity, reinforcing its role in autoimmune dysregulation. Interestingly, RBC count was demonstrated significant differences between patients in the active and inactive phases of TED. Subsequent Lasso regression and BSR regression analyses consistently identified RBC count as a robust predictor, demonstrating its significant association with TED clinical activity. RBCs are known to interact with various immune cells and participate in immune responses (30). They may affect immune homeostasis through triggering vascular endothelial dysregulation and altering complement-mediated immunomodulation (31). Another potential mechanism involves RBCs in oxygen transport and tissue oxygenation, autoregulating blood flow via S-nitroso-hemoglobin (SNO-Hb) -mediated nitric oxide (NO) bioactivity export (32). TED is characterized by orbital inflammation, edema, and increased fibrosis, which can lead to reduced blood flow and hypoxia in the affected tissues. A recent study revealed that hypoxia enhances the differentiation of Th1 cells, Th17 cells, and antigen-specific CD4^+^ cytotoxic T lymphocytes (CTLs), representing a key mechanistic pathway in T-cell-mediated TED pathogenesis (33). RBCs are responsible for delivering oxygen to tissues, and any alteration in RBC function or number may impact tissue oxygenation in the orbit. Therefore, RBCs may indirectly contribute to the development of TED by influencing the oxygenation status of orbital tissues.

By employing BSR and LASSO regressions for variable selection, this study constructed five predictive models. Among the five models, Model 1 exclusively incorporated imaging data, yielding a diagnostic efficacy of merely 0.7462. This suggests that relying solely on SPECT/CT imaging indicators is insufficient for evaluating active and inactive TED phases. Model 2, which integrates clinical characteristics with imaging data, demonstrated a significant enhancement in diagnostic performance (primary ROC: 0.832). However, five-fold cross-validation revealed moderate performance reduction (ROC mean: 0.7931), indicating potential sensitivity to data variability. Model 3, combining only clinical and blood markers, achieved a diagnostic efficiency exceeding 0.8 (primary ROC: 0.876). This model is particularly advantageous in primary hospitals lacking advanced detection equipment like SPECT/CT, as it still enables effective determination of TED phases. Notably, its cross-validated ROC mean (0.8196) showed greater divergence from the primary value, suggesting limited generalizability despite clinical utility. Model 4, which comprehensively combines clinical, imaging, and blood markers, achieved the highest diagnostic efficiency of 0.9118, indicating its superiority as a multidimensional model for assessing TED activity. Additionally, Model 4 demonstrated high calibration accuracy and the highest net benefit across a broad threshold range in DCA, suggesting its clinical utility in balancing sensitivity and specificity. Nevertheless, it exhibited the largest cross-validation discrepancy (ROC mean: 0.8196), likely reflecting overfitting from its broad variable set. This underscores the trade-off between model comprehensiveness and generalizability. In contrast, Model 5, constructed by intersecting univariate and multivariate significant variables (*p* < 0.05), demonstrated robust primary accuracy (AUC: 0.860) with superior stability during cross-validation (ROC mean: 0.8417). This optimized variable selection strategy effectively balanced discrimination power with generalizability, positioning it as a reliable pragmatic tool, particularly for rapid clinical assessments where resource efficiency is critical.

## Limitations

This study has some limitations. First, the limited sample size of 36 patients and the single-center retrospective nature might lead to selection bias and restrict the generalizability of the findings. Second, external validation in multicenter prospective cohorts is essential to confirm the model’s robustness and refine its predictive thresholds. Third, the biological mechanisms linking certain variables (e.g., RBC count, SBP) to TED activity remain unclear, necessitating mechanistic studies.

## Conclusion

This study developed a multidimensional predictive model for assessing clinical activity in TED by integrating clinical data, SPECT/CT imaging, and serum biomarkers. The concise and comprehensive Model 5, incorporating statistically screened predictors (sex, TRAb, EX, URmax, RBC), achieved balanced diagnostic accuracy with enhanced stability. This suggests that a multidimensional approach can provide a more holistic and precise assessment of TED activity than any single modality alone, offering a promising direction for improving clinical decision-making.

## Data Availability

The raw data supporting the conclusions of this article will be made available by the authors, without undue reservation.
